# Crystal structures of two magnesium citrates from powder diffraction data

**DOI:** 10.1107/S2056989020011913

**Published:** 2020-09-08

**Authors:** James A. Kaduk

**Affiliations:** a Department of Physics, North Central College, 131 S. Loomis, St., Naperville IL, 60540, USA; b Department of Chemistry, Illinois Institute of Technology, 3101 S. Dearborn St., Chicago IL 60616, USA

**Keywords:** powder diffraction, citrate ion, magnesium, density functional theory, Rietveld refinement

## Abstract

The crystal structures of magnesium hydrogen citrate dihydrate, Mg(HC_6_H_5_O_7_)(H_2_O)_2_, (I), and bis­(di­hydrogen citrato)magnesium, Mg(H_2_C_6_H_5_O_7_)_2_, (II), have been solved and refined using synchrotron X-ray powder diffraction data, and optimized using density functional techniques. In (I), the citrate anion occurs in the *trans, trans*-conformation, and triply chelates to the Mg cation. In (II), the citrate anion is *trans, gauche*, and doubly chelates to the Mg cation. In both compounds the Mg cation coordination polyhedron is an octa­hedron. In (I), the MgO_6_ coordination polyhedra are isolated, while in (II), they share edges to form chains. Strong O—H⋯O hydrogen bonds are prominent in the two structures, as well as in the previously reported magnesium citrate deca­hydrate.

## Abstract 2

The crystal structures of magnesium hydrogen citrate dihydrate and bis­(di­hydrogencitrato)magnesium have been solved and refined using synchrotron X-ray powder diffraction data, and optimized using density functional theory techniques.

## Chemical context   

A systematic study of the crystal structures of Group 1 (alkali metal) citrate salts has been reported in Rammohan & Kaduk (2018 ▸). This paper represents the extension of the study to Group 2 (alkaline earth) citrates. The only magnesium citrate previously reported is Mg_3_(C_6_H_5_O_7_)_2_(H_2_O)_10_, more properly formulated as [Mg(H_2_O)_6_][Mg(C_6_H_5_O_7_)(H_2_O)]_2_(H_2_O)_2_ (MGCITD; Johnson, 1965 ▸). I now describe the syntheses and crystal structures of magnesium hydrogen citrate dihydrate, Mg(HC_6_H_5_O_7_)(H_2_O)_2_ (I) and bis­(di­hydro­gen­citrato)magnesium, Mg(H_2_C_6_H_5_O_7_)_2_ (II). Attempts to prepare Be(H_2_C_6_H_5_O_7_)_2_, BeHC_6_H_5_O_7_, and Be_3_(C_6_H_5_O_7_)_2_ by HCl-catalyzed reaction of Be metal with a citric acid solution have so far yielded only amorphous products (see Fig. S1 in the supporting information).
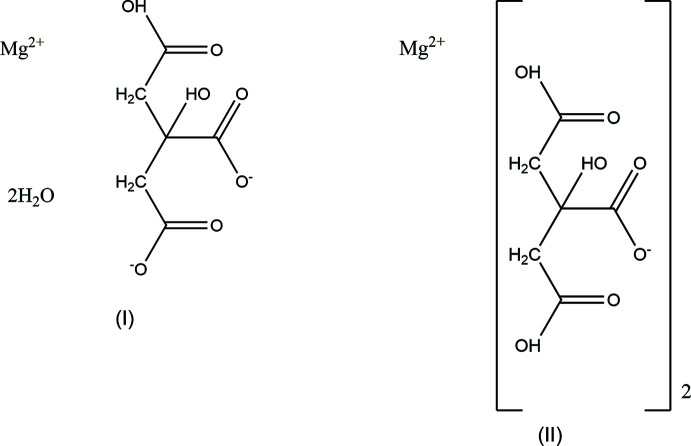


## Structural commentary   

**Figure 1:**
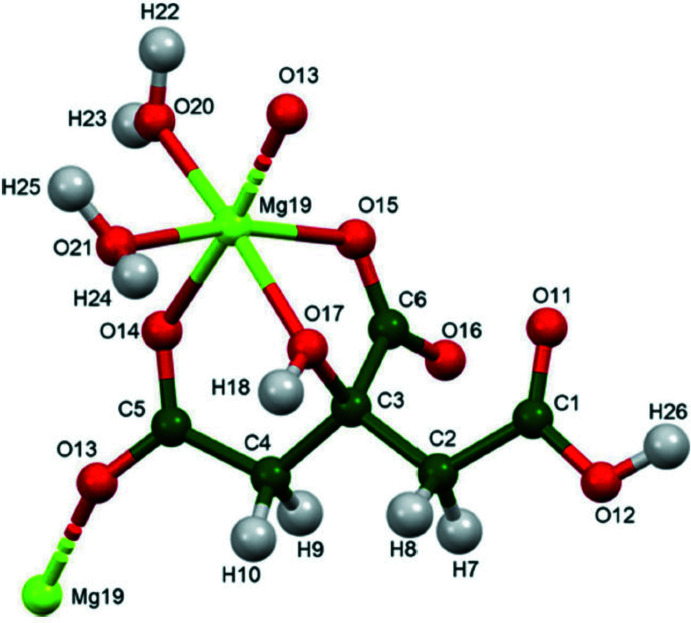
The expanded asymmetric unit of (I) with the atom numbering and 50% probability spheroids. Symmetry-generated atoms [Mg19(*x*, *y*, *z* − 1) and O13(*x*, *y*, *z* + 1)] are linked by dashed bonds.

**Figure 2:**
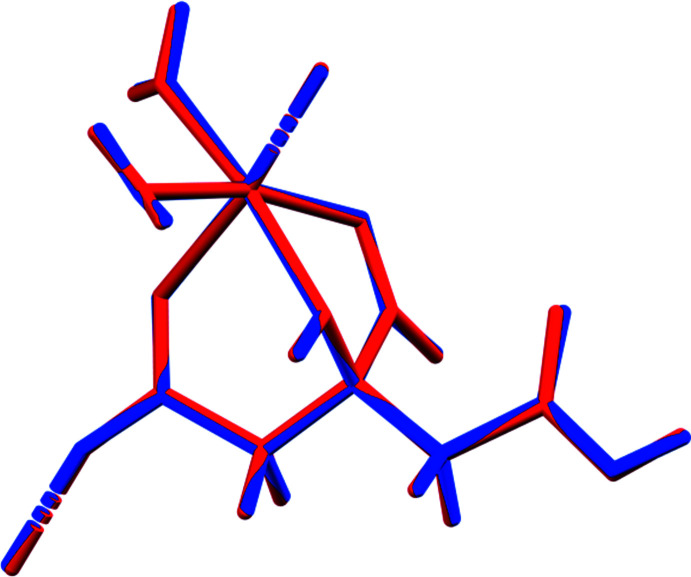
Comparison of the refined and optimized structures of (I). The refined structure is in red, and the DFT-optimized structure is in blue.

The crystal structure of (I) was solved and refined using synchrotron X-ray powder diffraction data, and optimized using density functional techniques. (Fig. 1 ▸) The root-mean-square Cartesian displacement of the non-hydrogen citrate atoms in the Rietveld refined and DFT-optimized structures is 0.062 Å (Fig. 2 ▸) The absolute difference in the position of the Mg cation in the unit cell is 0.055 Å. The excellent agreement between the structures is evidence that the experimental structure is correct (van de Streek & Neumann, 2014 ▸): the rest of the discussion will emphasize the DFT-optimized structure. All of the citrate bond distances, bond angles, and torsion angles fall within the normal ranges indicated by a *Mercury* Mogul geometry check (Macrae *et al.*, 2020 ▸). The citrate anion occurs in the *trans, trans*-conformation (about C2—C3 and C3—C4, respectively), which is one of the two low-energy conformations of an isolated citrate anion (Rammohan & Kaduk, 2018 ▸). The central carboxyl­ate group and the hydroxyl group exhibit a significant twist [O17—C3—C6—O15 = −15.6°] from the normal planar arrangement.

The Mg cation in (I) is six-coordinate (octa­hedral); the ligands are three carboxyl­ate oxygen atoms, the citrate hydroxyl group, and two *cis* water mol­ecules. The Mulliken overlap populations indicate that the Mg—O bonds have significant covalent character. The Mg bond-valence sum is 2.22. The citrate anion triply chelates to the Mg cation through the terminal carboxyl­ate O14, the central carboxyl­ate O15, and the hydroxyl group O17 oxygen atoms.

The Bravais–Friedel–Donnay–Harker (Bravais, 1866 ▸; Friedel, 1907 ▸; Donnay & Harker, 1937 ▸) method suggests that we might expect platy morphology for magnesium hydrogen citrate dihydrate, with {200} as the major faces. A 4th order spherical harmonic model was included in the refinement. The texture index was 1.000 (0), indicating that preferred orientation was not significant in this rotated capillary specimen.

**Figure 3:**
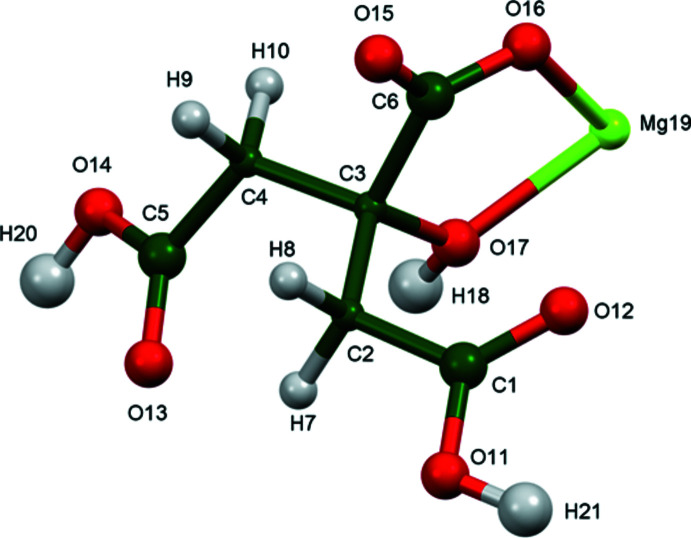
The asymmetric unit of (II) with the atom numbering and 50% probability spheroids.

**Figure 4:**
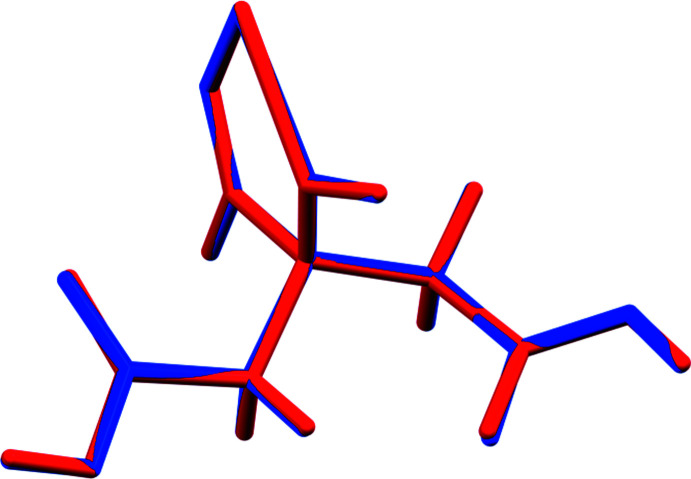
Comparison of the refined and optimized structures of (II). The refined structure is in red, and the DFT-optimized structure is in blue.

The crystal structure of (II) was solved and refined in the same way (Fig. 3 ▸) The root-mean-square Cartesian displacement of the non-hydrogen citrate atoms in the Rietveld refined and DFT-optimized structures is 0.043 Å (Fig. 4 ▸). The excellent agreement between the structures is evidence that the experimental structure is correct (van de Streek & Neumann, 2014 ▸) and this discussion will emphasize the DFT-optimized structure. All of the citrate bond distances, bond angles, and torsion angles fall within the normal ranges indicated by a *Mercury* Mogul geometry check (Macrae *et al.*, 2020 ▸). The citrate anion occurs in the *trans, gauche*-conformation (about C2—C3 and C3—C4, respectively), which is one of the two low-energy conformations of an isolated citrate anion (Rammohan & Kaduk, 2018 ▸). The central carboxyl­ate group and the hydroxyl group exhibit a significant twist [O17—C3—C6—O16 = 10.6°] from the normal planar arrangement.

The magnesium cation in (II) is six-coordinate (octa­hedral) and resides on a twofold axis; the ligands are two *cis* hydroxyl groups and 4 central carboxyl­ate groups O16. Ionizing the central carboxyl­ate group of citric acid first is the normal pattern (Rammohan & Kaduk, 2018 ▸). The Mulliken overlap populations indicate that the Mg—O bonds have significant covalent character and the Mg bond-valence sum is 2.12. The citrate anion doubly chelates to the Mg cation through the hydroxyl group O17 and the central carboxyl­ate group O16.

The Bravais–Friedel–Donnay–Harker method suggests that we might expect elongated morphology for crystals of (II), with [001] as the long axis. A 2nd order spherical harmonic model was included in the refinement. The texture index was 1.004 (0), indicating that preferred orientation was not significant in this rotated capillary specimen.

The root-mean-square Cartesian displacement of the non-hydrogen atoms in the reported and DFT-optimized structures of magnesium citrate deca­hydrate (MGCITD), [Mg(H_2_O)_6_][Mg(C_6_H_5_O_7_)(H_2_O)]_2_(H_2_O)_2_ are 0.016 Å for the *hexa*aqua cation and 0.030 Å for the citrate complex, confirming the excellent quality of the Johnson (1965 ▸) single-crystal structure. The citrate anion occurs in the *trans, trans* conformation. In Group 1 citrates, the *trans, gauche* conformation is more common for salts of the smaller alkali metals, and the *trans, trans* conformation is prevalent for the larger cations. Already with three Mg citrates, we see that the structures are more complicated. The torsion angle between the hydroxyl group and the central carboxyl­ate is only −4.8°. The citrate triply chelates to a Mg through the hydroxyl group, the central carboxyl­ate group, and one of the terminal carboxyl­ate groups.

## Supra­molecular features   

**Figure 5:**
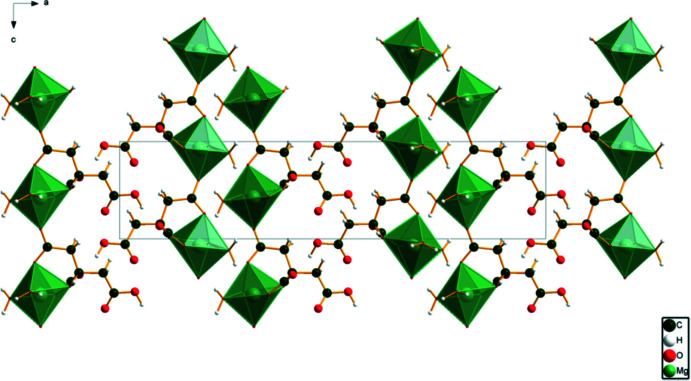
The crystal structure of (I), viewed down the *b* axis.

The MgO_6_ coordination polyhedra in (I) are isolated (Fig. 5 ▸). The crystal structure is characterized by layers parallel to the *bc*-plane. The un-ionized carb­oxy­lic acid O12—H26 forms a strong charge-assisted hydrogen bond to the central carboxyl­ate group O16. The hydroxyl group O17—H18 also acts as a donor to O16. All four protons of the water mol­ecules act as donors in O—H⋯O hydrogen bonds. Three of them involve ionized carboxyl­ate groups, and the fourth is to the other water mol­ecule. (Table 1 ▸).

**Figure 6:**
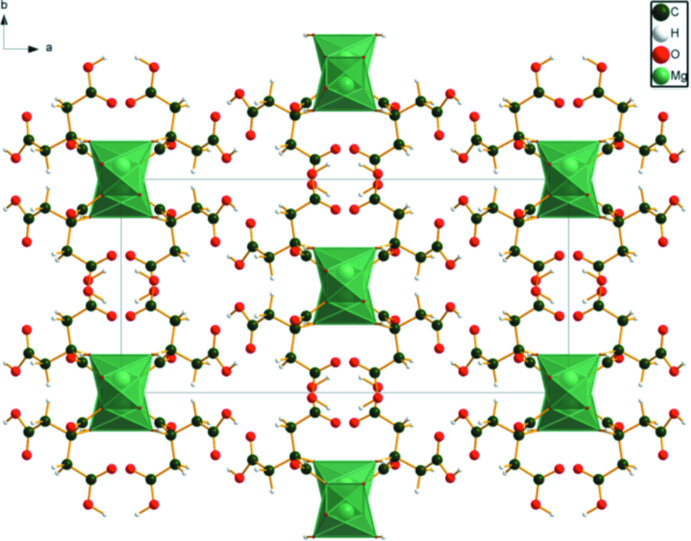
The crystal structure of (II), viewed down the *c* axis.

The MgO_6_ octa­hedra in (II) share edges to form chains propagating along the *c*-axis direction (Fig. 6 ▸). The two un-ionized terminal carb­oxy­lic acid groups form centrosymmetric 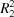
(8) loops, which link the citrate anions into chains along the *c*-axis direction. The hydroxyl group O17 forms an inter­molecular hydrogen bond to the central carboxyl­ate O15. The energies of the O—H⋯O hydrogen bonds were calculated using the correlation of Rammohan & Kaduk (2018 ▸). Weak C—H⋯O hydrogen bonds are also present (Table 2 ▸).

**Figure 7:**
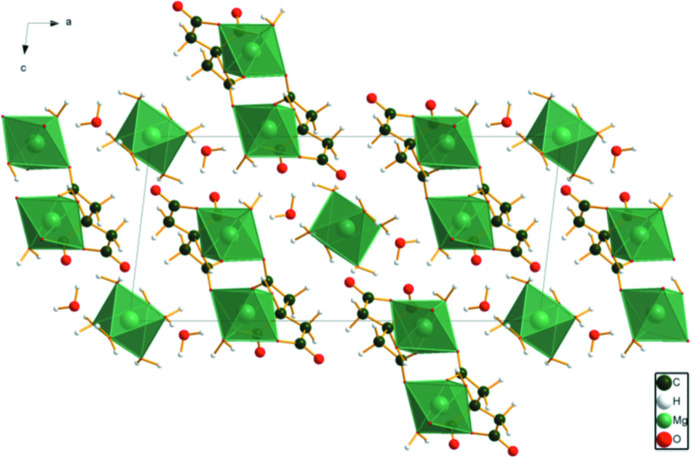
The crystal structure of [Mg(H_2_O)_6_][Mg(C_6_H_5_O_7_)(H_2_O)]_2_(H_2_O)_2_, (MGCITD) viewed down the *b* axis.

In magnesium citrate deca­hydrate (MGCITD), [Mg(H_2_O)_6_][Mg(C_6_H_5_O_7_)(H_2_O)]_2_(H_2_O)_2_, the MgO_6_ octa­hedra are isolated (Fig. 7 ▸). All of the H atoms of the water mol­ecules act as donors in O—H⋯O hydrogen bonds. The hydroxyl group forms bifurcated hydrogen bonds: one intra­molecular to the terminal carboxyl­ate O24 and the other inter­molecular to the terminal carboxyl­ate O28.

## Database survey   

Details of the comprehensive literature search for citrate structures are presented in Rammohan & Kaduk (2018 ▸). A search of the Cambridge Structural Database (Groom *et al.*, 2016 ▸) using a citrate fragment and Mg, C, H, and O only yielded Mg_3_(C_6_H_5_O_7_)_2_(H_2_O)_10_ (MGCITD; Johnson, 1965 ▸). Reduced-cell searches using the unit cells of both compounds of this study yielded no citrate structures. A search of the Powder Diffraction File (Gates-Rector & Blanton, 2019 ▸) yielded entry 02-063-3628 calculated from MGCITD, as well as the experimental entry 00-001-0186 (Hanawalt *et al.*, 1938 ▸) for the same compound.

## Synthesis and crystallization   

To prepare (I), magnesium hydrogen citrate dihydrate was synthesized by dissolving 2.0798 g (10.0 mmol) of H_3_C_6_H_5_O_7_(H_2_O) in 10 ml of water, and adding 0.8427 g (10.0 mmol) of ‘MgCO_3_’ to the clear solution [the magnesium carbonate reagent was actually Mg_5_(CO_3_)_4_(OH)_2_]. After slow fizzing, a clear colorless solution was obtained. This solution was dried in a 333 K oven to yield (I) as a white solid.

Compound (II) was obtained from the scale [94.5 (1) wt% magnesian calcite Ca_0.84_Mg_0.16_CO_3_, 5.3 (4) wt% brucite Mg(OH)_2_, and 0.2 (1) wt% vaterite polymorph of CaCO_3_] in a Megahome water still. The still was cleaned by filling the tank with tap water (from Lake Michigan), adding several tablespoons of citric acid monohydrate, and boiling for ∼2 h. The pale-yellow solution was deca­nted into a plastic pail, and allowed to evaporate at ambient conditions. Over five months, several white solids (calcium citrates, which will be discussed in another paper) crystallized, and were isolated. After five months, a clear yellow syrup remained. This was dried at 423 K to yield (II) as a white powder.

## Refinement   

Crystal data, data collection and structure refinement details for (I) are summarized in Table 3 ▸. A laboratory powder pattern, measured using Cu *K*α radiation, was indexed using *DICVOL* (Louër & Boultif, 2007 ▸) as incorporated into *FOX* (Favre-Nicolin & Černý, 2002 ▸) on a primitive ortho­rhom­bic cell with *a* = 26.9042 (24), *b* = 5.9323 (4), *c* = 6.1649 (5) Å, *V* = 985.27 (17) Å^3^, and *Z* = 4. Attempts to solve the structure with multiple programs using the laboratory data were unsuccessful. The powder pattern measured at 11-BM using a wavelength of 0.413070 Å was indexed on a primitive ortho­rhom­bic cell with *DICVOL* as incorporated into *FOX*: *a* = 26.91159 (14), *b* = 5.92442 (2), *c* = 6.15170 (2) Å, *V* = 980.800 (7) Å^3^, and *Z* = 4. The Space Group Explorer suggested *Pna2_1_*, which was confirmed by successful solution and refinement of the structure. The structure was solved using Monte Carlo-simulated annealing techniques as implemented in *FOX*. The scatterers were a citrate anion, a Mg atom, and two O atoms (water mol­ecules). In the best solution, one of the water mol­ecules was too close to a carboxyl­ate oxygen atom, and was discarded. The Mg coordination was 5/6 of an octa­hedron, so the second water mol­ecule was placed manually using *Materials Studio* (Dassault Systems, 2019 ▸).

**Figure 8:**
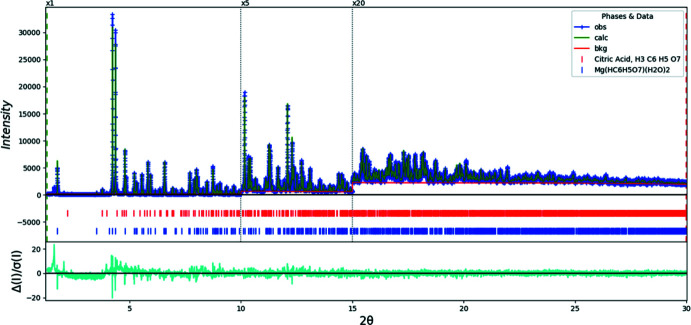
Rietveld plot for (I). The blue crosses represent the observed data points, and the green line is the calculated pattern. The cyan curve is the normalized error plot. The vertical scale has been multiplied by a factor of 5× for 2θ > 10.0°, and by a factor of 20× for 2θ > 15.0°. The row of blue tick marks indicates the calculated reflection positions, and the red tick marks indicate the peak positions for the citric acid impurity. The red line is the background curve.

The structure of (I) was refined by the Rietveld method using *GSAS-II* (Toby & Von Dreele, 2013 ▸) (Fig. 8 ▸). The initial refinement clarified the presence of extra peaks, which were identified as citric acid (02-061-2110; CITRAC10), which was added as a second phase; its concentration refined to 12.2 wt%. A few very weak peaks indicate the presence of an unidentified impurity. Analysis of potential hydrogen bonding using *Mercury* (Macrae *et al.*, 2020 ▸) made it possible to determine approximate positions for the hydroxyl hydrogen atom H18 and the four water mol­ecule hydrogen atoms. The C1—O12 bond was longer than the other carboxyl­ate distances, and the O12⋯O16^i^ distance was 2.62 Å, making it clear that H26, the proton of the un-ionized carboxyl group, was located on O12. All heavy-atom bond distances and angles of the citrate anion were restrained: C1—C2 = C4—C5 = 1.51 (3), C2—C3 = C3—C4 = 1.54 (3), C3—C6 = 1.55 (3), C3—O17 = 1.42 (3), C1—O11 = 1.22 (3), C1—O12 = 1.32 (3), and the C—O of the ionized carboxyl­ate groups = 1.27 (3) Å, C1—C2—C3 = C3—C4—C5 = 115 (3), the angles around C3 = 109.5 (3), the O—C—C angles of the carboxyl­ate groups = 115 (3), and the O—C—O angles of the carboxyl­ate groups = 130 (3)°. The restraints contributed 1.5% to the final χ^2^. The hydrogen atoms were included in fixed positions, which were re-calculated during the course of the refinement using *Materials Studio*. The *U*_iso_ values of C2, C3, and C4 were constrained to be equal, and those of H7, H8, H9, and H10 were constrained to be 1.3× that of these carbon atoms. The *U*_iso_ values of C1, C5, C6, and the oxygen atoms were constrained to be equal, and that of H18 was constrained to be 1.3× this value. The *U*_iso_ values of the O atoms of the water mol­ecules were constrained to be equal, and the *U*_iso_ values of their H atoms to be 1.3× this value. The background was described by a four-term shifted Chebyshev polynomial, with a peak at 10.84° to describe the scattering from the Kapton capillary and any amorphous component.

A density functional geometry optimization for (I) (fixed experimental unit cell) was carried out using *CRYSTAL09* (Dovesi *et al.*, 2005 ▸). The basis sets for the H, C, N, and O atoms were those of Gatti *et al.* (1994 ▸), and the basis set for Mg was that of McCarthy & Harrison (1994 ▸). The calculation used 8 *k*-points and the B3LYP functional, and took around four days on a 2.4 GHz PC.

Crystal data, data collection and structure refinement details for (II) are summarized in Table 3 ▸. It proved difficult to index the laboratory pattern, though the correct cell was included in hits found by *DICVOL06* (Louër & Boultif, 2007 ▸). The synchrotron pattern was indexed on a primitive monoclinic unit cell with *N-TREOR* (Altomare *et al.*, 2013 ▸): *a* = 23.24984 (8), *b* = 10.97779 (3), *c* = 5.92449 (1) Å, *β* = 979.1860 (2)°, *V* = 1500.241 (8) Å^3^, and *Z* = 4. The systematic absences unambiguously determined the space group as *P2_1_/c* The structure was solved by direct methods using *EXPO2009* (Altomare *et al.*, 2013 ▸), assuming that it was a Ca salt. During the refinement, the electron density at the metal site and the metal–oxygen bond distances made it clear that it was a Mg salt rather than a Ca compound.

**Figure 9:**
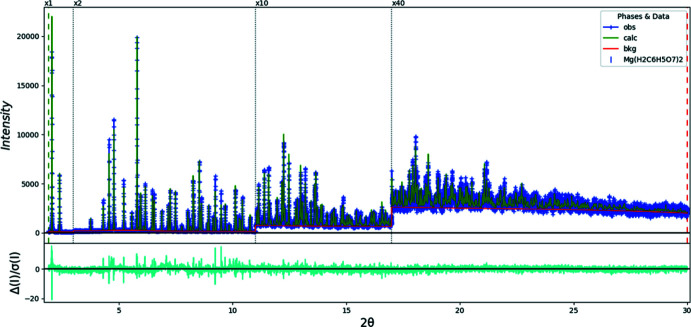
Rietveld plot for (II). The blue crosses represent the observed data points, and the green line is the calculated pattern. The cyan curve is the normalized error plot. The vertical scale has been multiplied by a factor of 2× for 2θ > 3.0°, by a factor of 10× for 2θ > 12.0°, and by a factor of 40× for 2θ > 17.0°. The row of blue tick marks indicates the calculated reflection positions. The red line is the background curve.

The structure was refined by the Rietveld method using *GSAS-II* (Toby & Von Dreele, 2013 ▸) (Fig. 9 ▸). Analysis of the refined structure using *PLATON* (Spek, 2020 ▸) and the Find Symmetry module of *Materials Studio* (Dassault Systems, 2019 ▸) suggested the presence of extra symmetry, and that the true space group was *C2/c* (transformation matrix 1 0 1 / 0 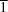 0 / 0 0 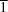). The structure was re-refined in this space group, using the strategy described above for (I). The position of the peak in the background was 5.37°.

A density functional geometry optimization for (II) (fixed experimental unit cell) was carried out using *CRYSTAL17* (Dovesi *et al.*, 2018 ▸). The basis sets for the H, C, N, and O atoms were those of Gatti *et al.* (1994 ▸), and the basis set for Mg was that of Peintinger *et al.* (2013 ▸). The calculation used 8 *k*-points and the B3LYP functional, and took ∼15 h on a 3.54 GHz PC.

A density functional geometry optimization (fixed experimental unit cell) of the structure of magnesium citrate deca­hydrate (MGCITD) was carried out using *CRYSTAL09* (Dovesi *et al.*, 2005 ▸). The basis sets for the H, C, N, and O atoms were those of Gatti *et al.* (1994 ▸), and the basis set for Mg was that of McCarthy & Harrison (1994 ▸). The calculation used 8 *k*-points and the B3LYP functional, and took 11 days on a 2.4 GHz PC.

## Supplementary Material

Crystal structure: contains datablock(s) global, II, MGCITD_DFT, I_overall, I, ramm026_11bm_pwd_0, I_impurity, I_DFT, II_DFT. DOI: 10.1107/S2056989020011913/hb7927sup1.cif

Powder patters of beryllium citrates. DOI: 10.1107/S2056989020011913/hb7927sup2.docx

CCDC references: 2026313, 2026312, 2026311, 2026310, 2026309, 2026308

Additional supporting information:  crystallographic information; 3D view; checkCIF report

## Figures and Tables

**Table 1:** Hydrogen-bond geometry (Å, °) for (I) (DFT)

*D*—H⋯*A*	*D*—H	H⋯*A*	*D*⋯*A*	*D*—H⋯*A*
O12—H26⋯O16^i^	1.00	1.64	2.614	161
O17—H18⋯O16^ii^	1.00	1.69	2.682	176
O20—H22⋯O21^iii^	0.98	1.82	2.795	171
O20—H23⋯O13^iii^	0.98	1.89	2.844	166
O21—H24⋯O15^ii^	1.00	1.69	2.666	166
O21—H25⋯O14^iv^	0.99	1.81	2.792	174

Symmetry codes: (i) 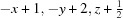; (ii) 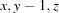; (iii) 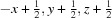; (iv) 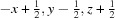.

**Table 2:** Hydrogen-bond geometry (Å, °) for (II) (DFT)

*D*—H⋯*A*	*D*—H	H⋯*A*	*D*⋯*A*	*D*—H⋯*A*
O11—H21⋯O12^i^	1.02	1.55	2.567	179
O14—H20⋯O13^ii^	1.01	1.64	2.640	176
O17—H18⋯O15^iii^	0.99	1.72	2.708	174
C4—H9⋯O13^iv^	1.10	2.57	3.580	152
C4—H10⋯O15^v^	1.09	2.47	3.522	161

Symmetry codes: (i) 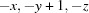; (ii) 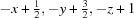; (iii) 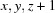; (iv) 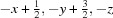; (v) 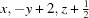.

**Table 3:** Experimental details

	(I)	(II)
Crystal data		
Chemical formula	Mg^2+^·C_6_H_6_O_7_^2−^·2H_2_O	Mg(H_2_C_6_H_5_O_7_)_2_
*M*_r_	250.44	380.13
Crystal system, space group	Orthorhombic, *P* *n* *a*2_1_	Monoclinic, *C*2/*c*
Temperature (K)	295	295
*a*, *b*, *c* (Å)	26.91181 (13), 5.924517 (17), 6.151787 (18)	23.26381 (16), 10.97790 (4), 5.924466 (18)
α, β, γ (°)	90, 90, 90	90, 82.5511 (3), 90
*V* (Å^3^)	980.84 (1)	1500.267 (6)
*Z*	4	4
Radiation type	Synchrotron, λ = 0.41307 Å	Synchrotron, λ = 0.41307 Å
Specimen shape, size (mm)	Cylinder, 3.0 × 1.5	Cylinder, 3.0 × 1.5

Data collection		
Diffractometer	APS 11-BM	11-BM APS
Specimen mounting	Kapton capillary	Kapton capillary
Data collection mode	Transmission	Transmission
Data collection method	Step	Step
θ values (°)	2θ_min_ = 0.500 2θ_max_ = 49.991 2θ_step_ = 0.001	2θ_min_ = 0.500 2θ_max_ = 49.991 2θ_step_ = 0.001

Refinement		
*R* factors and goodness of fit	*R*_p_ = 0.086, *R*_wp_ = 0.110, *R*_exp_ = 0.060, χ^2^ = 3.486	*R*_p_ = 0.098, *R*_wp_ = 0.120, *R*_exp_ = 0.083, χ^2^ = 2.16
No. of parameters	76	60
No. of restraints	29	–

Computer programs: *FOX* (Favre-Nicolin & Černý, 2002 ▸), *GSAS-II* (Toby & Von Dreele, 2013 ▸), *Mercury* (Macrae *et al.*, 2020 ▸), *DIAMOND* (Crystal Impact, 2015 ▸), *publCIF* (Westrip, 2010 ▸).

## supplementary crystallographic information

### magnesium hydrogen citrate dihydrate (I) Crystal data

**Table:**

Mg^2+^·C_6_H_6_O_7_^2^^−^·2H_2_O	*V* = 980.84 (1) Å^3^
*M**_r_* = 250.44	*Z* = 4
Orthorhombic, *P**n**a*2_1_	*D*_x_ = 1.696 Mg m^−^^3^
*a* = 26.91181 (13) Å	Synchrotron radiation
*b* = 5.924517 (17) Å	*T* = 295 K
*c* = 6.151787 (18) Å	cylinder, 3.0 × 1.5 mm

### magnesium hydrogen citrate dihydrate (I) Data collection

**Table:**

APS 11-BM diffractometer	Data collection mode: transmission
Specimen mounting: Kapton capillary	Scan method: step

### magnesium hydrogen citrate dihydrate (I) Refinement

**Table:**

Profile function: Crystallite size in microns with "isotropic" model: parameters: Size, G/L mix 1.000, 1.000, Microstrain, "generalized" model (10^6^ * delta Q/Q) parameters: S400, S040, S004, S220, S202, S022, G/L mix 19.594, 2586.054, 2128.049, 48.827, -44.218, 6452.631, 1.000,	Preferred orientation correction: Simple spherical harmonic correction Order = 2 Coefficients: 0:0:C(2,0) = 0.025(4); 0:0:C(2,2) = -0.025(5)
29 restraints	

### magnesium hydrogen citrate dihydrate (I) Fractional atomic coordinates and isotropic or equivalent isotropic displacement parameters (Å^2^)

**Table:**

	*x*	*y*	*z*	*U*_iso_*/*U*_eq_	
C1	0.48943 (13)	0.7789 (8)	0.55610	0.0202 (4)*	
C2	0.46229 (15)	0.7167 (7)	0.3481 (7)	0.0103 (9)*	
C3	0.40501 (13)	0.7286 (6)	0.3574 (9)	0.0103*	
C4	0.38562 (14)	0.7010 (7)	0.1229 (9)	0.0103*	
C5	0.32959 (12)	0.6702 (9)	0.0955 (9)	0.0202*	
C6	0.38764 (16)	0.9591 (6)	0.4509 (10)	0.0202*	
H7	0.47783	0.80849	0.21591	0.0133*	
H8	0.47190	0.53629	0.31678	0.0133*	
H9	0.39783	0.84023	0.00853	0.0133*	
H10	0.40434	0.55040	0.03933	0.0133*	
O11	0.46605 (12)	0.8354 (7)	0.7185 (7)	0.0202*	
O12	0.53720 (11)	0.7677 (6)	0.5337 (8)	0.0202*	
O13	0.31529 (12)	0.6138 (6)	−0.0898 (9)	0.0202*	
O14	0.30170 (12)	0.6980 (6)	0.2576 (9)	0.0202*	
O15	0.35114 (12)	0.9501 (5)	0.5750 (9)	0.0202*	
O16	0.40759 (12)	1.1313 (6)	0.3708 (9)	0.0202*	
O17	0.38526 (12)	0.5579 (5)	0.4951 (9)	0.0202*	
H18	0.39334	0.40298	0.44299	0.0263*	
Mg19	0.31438 (7)	0.6445 (3)	0.5864 (8)	0.0152 (5)*	
O20	0.24223 (12)	0.7558 (5)	0.6155 (10)	0.0183 (8)*	
O21	0.29100 (11)	0.3095 (5)	0.5592 (9)	0.0183*	
H22	0.22860	0.77371	0.75942	0.0238*	
H23	0.22652	0.87428	0.52100	0.0238*	
H24	0.31432	0.18972	0.57251	0.0238*	
H25	0.25819	0.26280	0.61541	0.0238*	
H26	0.55280	0.81813	0.67454	0.0263*	

### magnesium hydrogen citrate dihydrate (I) Geometric parameters (Å, º)

**Table:**

C1—C2	1.519 (4)	O13—C5	1.249 (4)
C1—O11	1.227 (4)	O13—Mg19^i^	2.000 (4)
C1—O12	1.294 (3)	O14—C5	1.259 (4)
C2—C1	1.519 (4)	O14—Mg19	2.075 (4)
C2—C3	1.544 (4)	O15—C6	1.245 (4)
C2—H7	1.064	O15—Mg19	2.065 (3)
C2—H8	1.117	O16—C6	1.254 (4)
C3—C2	1.544 (4)	O17—C3	1.423 (4)
C3—C4	1.543 (4)	O17—H18	0.996
C3—C6	1.553 (4)	O17—Mg19	2.054 (4)
C3—O17	1.423 (4)	H18—O17	0.996 (3)
C4—C3	1.543 (4)	Mg19—O13^ii^	2.000 (4)
C4—C5	1.528 (4)	Mg19—O14	2.075 (4)
C4—H9	1.133	Mg19—O15	2.065 (3)
C4—H10	1.147	Mg19—O17	2.054 (4)
C5—C4	1.528 (4)	Mg19—O20	2.059 (4)
C5—O13	1.249 (4)	Mg19—O21	2.089 (3)
C5—O14	1.259 (4)	O20—Mg19	2.059 (4)
C6—C3	1.553 (4)	O20—H22	0.964
C6—O15	1.245 (4)	O20—H23	1.005
C6—O16	1.254 (4)	O21—Mg19	2.089 (3)
H7—C2	1.064	O21—H24	0.9507
H8—C2	1.117	O21—H25	0.988
H9—C4	1.133	H22—O20	0.964
H10—C4	1.147	H23—O20	1.005
O11—C1	1.227 (4)	H24—O21	0.950
O12—C1	1.294 (3)	H25—O21	0.988
O12—H26	1.008	H26—O12	1.008
			
C2—C1—O11	120.4 (3)	C5—O13—Mg19^i^	152.7 (4)
C2—C1—O12	112.0 (3)	C5—O14—Mg19	130.8 (3)
O11—C1—O12	127.6 (3)	C6—O15—Mg19	115.9 (3)
C1—C2—C3	115.9 (3)	C3—O17—H18	112.5
C1—C2—H7	109.3	C3—O17—Mg19	109.4 (2)
C3—C2—H7	113.4	H18—O17—Mg19	121.41
C1—C2—H8	105.4	O13^ii^—Mg19—O14	170.49 (17)
C3—C2—H8	106.3	O13^ii^—Mg19—O15	96.18 (18)
H7—C2—H8	105.5	O14—Mg19—O15	84.94 (17)
C2—C3—C4	107.3 (3)	O13^ii^—Mg19—O17	103.79 (17)
C2—C3—C6	110.8 (3)	O14—Mg19—O17	85.67 (16)
C4—C3—C6	109.7 (3)	O15—Mg19—O17	76.40 (14)
C2—C3—O17	111.3 (3)	O13^ii^—Mg19—O20	87.36 (17)
C4—C3—O17	110.8 (3)	O14—Mg19—O20	83.15 (17)
C6—C3—O17	107.0 (3)	O15—Mg19—O20	100.02 (15)
C3—C4—C5	116.7 (3)	O17—Mg19—O20	168.5 (2)
C3—C4—H9	113.9	O13^ii^—Mg19—O21	89.83 (17)
C5—C4—H9	107.7	O14—Mg19—O21	91.01 (19)
C3—C4—H10	110.7	O15—Mg19—O21	167.16 (18)
C5—C4—H10	106.9	O17—Mg19—O21	91.17 (15)
H9—C4—H10	99.2	O20—Mg19—O21	91.56 (15)
C4—C5—O13	115.9 (3)	Mg19—O20—H22	118.30
C4—C5—O14	119.0 (3)	Mg19—O20—H23	124.8
O13—C5—O14	125.1 (3)	H22—O20—H23	107.1
C3—C6—O15	115.3 (3)	Mg19—O21—H24	120.21
C3—C6—O16	116.2 (3)	Mg19—O21—H25	120.48
O15—C6—O16	127.8 (3)	H24—O21—H25	110.6
C1—O12—H26	107.9		

Symmetry codes: (i) *x*, *y*, *z*−1; (ii) *x*, *y*, *z*+1.

### (I_DFT) Crystal data

**Table:**

C_6_H_10_MgO_9_	*b* = 5.9244 Å
*M**_r_* = 250.44	*c* = 6.1517 Å
Orthorhombic, *P**n**a*2_1_	*V* = 980.80 Å^3^
*a* = 26.9116 Å	*Z* = 4

### (I_DFT) Data collection

**Table:**

*h* = →	*l* = →
*k* = →	

### (I_DFT) Fractional atomic coordinates and isotropic or equivalent isotropic displacement parameters (Å^2^)

**Table:**

	*x*	*y*	*z*	*U*_iso_*/*U*_eq_	
C1	0.48690	0.78384	0.55610	0.03000*	
C2	0.46156	0.71131	0.34896	0.03000*	
C3	0.40476	0.72893	0.34810	0.03000*	
C4	0.38635	0.69837	0.11128	0.03000*	
C5	0.33073	0.66732	0.08379	0.03000*	
C6	0.38608	0.95837	0.43867	0.03000*	
H7	0.47783	0.80849	0.21591	0.039000*	
H8	0.47190	0.53629	0.31678	0.039000*	
H9	0.39783	0.84023	0.00853	0.039000*	
H10	0.40434	0.55040	0.03933	0.039000*	
O11	0.46603	0.86068	0.71575	0.03000*	
O12	0.53597	0.75370	0.54317	0.03000*	
O13	0.31511	0.59826	−0.09886	0.03000*	
O14	0.30103	0.70296	0.24037	0.03000*	
O15	0.34967	0.95455	0.57014	0.03000*	
O16	0.40589	1.13578	0.36647	0.03000*	
O17	0.38352	0.55877	0.48700	0.03000*	
H18	0.39334	0.40298	0.44299	0.039000*	
Mg19	0.31266	0.64580	0.57726	0.03000*	
O20	0.24313	0.76390	0.61354	0.03000*	
O21	0.28934	0.31025	0.54282	0.03000*	
H22	0.22860	0.77371	0.75942	0.039000*	
H23	0.22652	0.87428	0.52100	0.039000*	
H24	0.31432	0.18972	0.57251	0.039000*	
H25	0.25819	0.26280	0.61541	0.039000*	
H26	0.55280	0.81813	0.67454	0.039000*	

### (I_DFT) Hydrogen-bond geometry (Å, º)

**Table:**

*D*—H···*A*	*D*—H	H···*A*	*D*···*A*	*D*—H···*A*
O12—H26···O16^i^	1.00	1.64	2.614	161
O17—H18···O16^ii^	1.00	1.69	2.682	176
O20—H22···O21^iii^	0.98	1.82	2.795	171
O20—H23···O13^iii^	0.98	1.89	2.844	166
O21—H24···O15^ii^	1.00	1.69	2.666	166
O21—H25···O14^iv^	0.99	1.81	2.792	174

Symmetry codes: (i) −*x*+1, −*y*+2, *z*+1/2; (ii) *x*, *y*−1, *z*; (iii) −*x*+1/2, *y*+1/2, *z*+1/2; (iv) −*x*+1/2, *y*−1/2, *z*+1/2.

### (I_impurity) Crystal data

**Table:**

C_6_H_8_O_7_	β = 111.2291 (14)°
*M**_r_* = 192.12	*V* = 770.06 (2) Å^3^
Monoclinic, *P*2_1_/*a*	*Z* = 4
*a* = 12.8139 (7) Å	*D*_x_ = 1.657 Mg m^−^^3^
*b* = 5.62177 (11) Å	*T* = 295 K
*c* = 11.4681 (6) Å	

### (I_impurity) Refinement

**Table:**

Profile function: Crystallite size in microns with "isotropic" model: parameters: Size, G/L mix 1.000, 1.000, Microstrain, "isotropic" model (10^6^ * delta Q/Q) parameters: Mustrain, G/L mix 2.57(4)e3, 1.000,	Preferred orientation correction: March-Dollase correction coef. = 1.000 axis = [0, 0, 1]

### (I_impurity) Fractional atomic coordinates and isotropic or equivalent isotropic displacement parameters (Å^2^)

**Table:**

	*x*	*y*	*z*	*U*_iso_*/*U*_eq_	
C1	0.08920	−0.54280	0.39950	0.025*	
C2	0.15990	−0.56050	0.32070	0.025*	
C3	0.16260	−0.80550	0.26120	0.025*	
C4	0.24750	−0.79220	0.19560	0.025*	
C5	0.26980	−1.03280	0.15150	0.025*	
C6	0.04560	−0.86720	0.16750	0.025*	
H1	0.13730	−0.43320	0.25620	0.025*	
H2	0.23180	−0.52200	0.37470	0.025*	
H3	0.22140	−0.68820	0.12140	0.025*	
H4	0.31830	−0.72800	0.25370	0.025*	
H5	0.02820	−0.31480	0.49180	0.025*	
H6	0.38460	−1.22410	0.16410	0.025*	
H7	−0.05570	−0.76850	0.01350	0.025*	
H8	0.14360	−1.03130	0.36590	0.025*	
O1	0.07850	−0.32830	0.43520	0.025*	
O2	0.04560	−0.71880	0.42820	0.025*	
O3	0.37730	−1.07600	0.18790	0.025*	
O4	0.19930	−1.17200	0.09030	0.025*	
O5	0.01470	−0.72840	0.06840	0.025*	
O6	−0.01090	−1.02400	0.18460	0.025*	
O7	0.20030	−0.98600	0.35170	0.025*	

### (I_impurity) Geometric parameters (Å, º)

**Table:**

C1—C2	1.4967	H1—H2	1.5397
C1—O1	1.2966	H2—C2	0.9306
C1—O2	1.2380	H2—H1	1.5397
C2—C1	1.4967	H3—C4	0.9857
C2—C3	1.5430	H3—H4	1.5904
C2—H1	0.994	H4—C4	0.9795
C2—H2	0.9306	H4—H3	1.5904
C3—C2	1.5430	H5—O1	1.0703
C3—C4	1.5322	H6—O3	0.8916
C3—C6	1.5349	H7—O5	0.9218
C3—O7	1.4061	H8—O7	0.8396
C4—C3	1.5322	O1—C1	1.2966
C4—C5	1.5072	O1—H5	1.0703
C4—H3	0.9857	O2—C1	1.2380
C4—H4	0.9795	O3—C5	1.3093
C5—C4	1.5072	O3—H6	0.8916
C5—O3	1.3093	O4—C5	1.2091
C5—O4	1.2091	O5—C6	1.3158
C6—C3	1.5349	O5—H7	0.9218
C6—O5	1.3158	O6—C6	1.2010
C6—O6	1.2010	O7—C3	1.4061
H1—C2	0.994	O7—H8	0.8396
			
C2—C1—O1	114.13	C3—C4—H3	111.932
C2—C1—O2	122.512	C5—C4—H3	106.793
O1—C1—O2	123.357	C3—C4—H4	109.352
C1—C2—C3	116.215	C5—C4—H4	108.511
C1—C2—H1	108.935	H3—C4—H4	108.048
C3—C2—H1	111.145	C4—C5—O3	111.401
C1—C2—H2	104.631	C4—C5—O4	125.664
C3—C2—H2	109.06	O3—C5—O4	122.935
H1—C2—H2	106.214	C3—C6—O5	112.508
C2—C3—C4	107.888	C3—C6—O6	123.044
C2—C3—C6	109.796	O5—C6—O6	124.445
C4—C3—C6	110.76	C1—O1—H5	114.406
C2—C3—O7	111.993	C5—O3—H6	106.888
C4—C3—O7	106.402	C6—O5—H7	111.033
C6—C3—O7	109.943	C3—O7—H8	105.778
C3—C4—C5	112.058		

### Magnesium bis(dihydrogen citrate) (II) Crystal data

**Table:**

Mg^2+^·2C_6_H_7_O_7_^−^	*V* = 1500.27 (1) Å^3^
*M**_r_* = 406.53	*Z* = 4
Monoclinic, *C*2/*c*	*D*_x_ = 1.800 Mg m^−^^3^
*a* = 23.26381 (16) Å	Synchrotron radiation, λ = 0.41307 Å
*b* = 10.97790 (4) Å	*T* = 295 K
*c* = 5.924466 (18) Å	white
β = 82.5511 (3)°	cylinder, 3.0 × 1.5 mm

### Magnesium bis(dihydrogen citrate) (II) Data collection

**Table:**

APS 11-BM diffractometer	Scan method: step
Specimen mounting: Kapton capillary	2θ_min_ = 0.500°, 2θ_max_ = 49.991°, 2θ_step_ = 0.001°
Data collection mode: transmission	

### Magnesium bis(dihydrogen citrate) (II) Refinement

**Table:**

Least-squares matrix: full	Profile function: Finger-Cox-Jephcoat function parameters U, V, W, X, Y, SH/L: peak variance(Gauss) = Utan(Th)^2^+Vtan(Th)+W: peak HW(Lorentz) = X/cos(Th)+Ytan(Th); SH/L = S/L+H/L U, V, W in (centideg)^2^, X & Y in centideg 1.163, -0.126, 0.063, 0.000, 0.000, 0.002, Crystallite size in microns with "isotropic" model: parameters: Size, G/L mix 1.000, 1.000, Microstrain, "uniaxial" model (10^6^ * delta Q/Q) anisotropic axis is [0, 0, 1] parameters: equatorial mustrain, axial mustrain, G/L mix 1556(11), 880(8), 1.000,
*R*_p_ = 0.098	60 parameters
*R*_wp_ = 0.120	H-atom parameters not defined?
*R*_exp_ = 0.083	(Δ/σ)_max_ = 2.829
*R*(*F*^2^) = 0.08746	Background function: Background function: "chebyschev-1" function with 4 terms: 75.08(16), -28.81(26), 8.70(18), -4.70(14), Background peak parameters: pos, int, sig, gam: 5.370(17), 7.52(18)e4, 6.02(27)e3, 0.100,
49492 data points	Preferred orientation correction: Simple spherical harmonic correction Order = 2 Coefficients: 0:0:C(2,-2) = -0.0720(26); 0:0:C(2,0) = 0.039(4); 0:0:C(2,2) = 0.1089(29)

### Magnesium bis(dihydrogen citrate) (II) Fractional atomic coordinates and isotropic or equivalent isotropic displacement parameters (Å^2^)

**Table:**

	*x*	*y*	*z*	*U*_iso_*/*U*_eq_	
C1	0.06727 (13)	0.5905 (3)	0.0107 (8)	0.0280 (4)*	
C2	0.12138 (12)	0.6630 (3)	0.0408 (6)	0.0135 (7)*	
C3	0.11122 (10)	0.7998 (2)	0.0807 (5)	0.0135*	
C4	0.16939 (14)	0.8630 (3)	0.0995 (5)	0.0135*	
C5	0.20688 (16)	0.8168 (3)	0.2773 (6)	0.0280*	
C6	0.08530 (15)	0.8589 (3)	−0.1256 (5)	0.0280*	
H7	0.14177	0.62313	0.18170	0.0176*	
H8	0.14929	0.64867	−0.11299	0.0176*	
H9	0.19616	0.85871	−0.06251	0.0176*	
H10	0.16162	0.96177	0.13684	0.0176*	
O11	0.07343 (11)	0.4733 (2)	0.0383 (5)	0.0280*	
O12	0.02252 (12)	0.6459 (2)	−0.0215 (5)	0.0280*	
O13	0.20723 (12)	0.7082 (2)	0.3239 (4)	0.0280*	
O14	0.23727 (12)	0.9021 (2)	0.3559 (5)	0.0280*	
O15	0.11197 (12)	0.8383 (2)	−0.3181 (5)	0.0280*	
O16	0.04262 (12)	0.9288 (2)	−0.0762 (4)	0.0280*	
O17	0.07035 (10)	0.8186 (2)	0.2785 (4)	0.0280*	
H18	0.08676	0.82392	0.42629	0.0364*	
Mg19	0.00000	0.93974 (19)	0.25000	0.0202 (7)*	
H20	0.25763	0.86212	0.48563	0.0364*	
H21	0.03540	0.42942	0.03325	0.0364*	

### Magnesium bis(dihydrogen citrate) (II) Geometric parameters (Å, º)

**Table:**

C1—C2	1.519 (3)	H10—C4	1.1165
C1—O11	1.308 (3)	O11—C1	1.308 (3)
C1—O12	1.241 (3)	O11—H21	1.011 (3)
C2—C1	1.519 (3)	O12—C1	1.241 (3)
C2—C3	1.534 (3)	O13—C5	1.224 (3)
C2—H7	1.1030	O14—C5	1.296 (3)
C2—H8	1.060	O14—H20	1.0500
C3—C2	1.534 (3)	O15—C6	1.247 (3)
C3—C4	1.538 (3)	O16—C6	1.259 (3)
C3—C6	1.571 (3)	O16—Mg19	2.058 (3)
C3—O17	1.425 (3)	O16—Mg19^i^	2.096 (3)
C4—C3	1.538 (3)	O17—C3	1.425 (3)
C4—C5	1.538 (3)	O17—H18	1.0013
C4—H9	1.076	O17—Mg19	2.133 (3)
C4—H10	1.1165	H18—O17	1.0013
C5—C4	1.538 (3)	Mg19—O16	2.058 (3)
C5—O13	1.224 (3)	Mg19—O16^ii^	2.058 (3)
C5—O14	1.296 (3)	Mg19—O16^i^	2.096 (3)
C6—C3	1.571 (3)	Mg19—O16^iii^	2.096 (3)
C6—O15	1.247 (3)	Mg19—O17	2.133 (3)
C6—O16	1.259 (3)	Mg19—O17^ii^	2.133 (3)
H7—C2	1.1030	H20—O14	1.0500
H8—C2	1.060	H21—O11	1.0110
H9—C4	1.076		
			
C2—C1—O11	113.3 (2)	H9—C4—H10	106.22
C2—C1—O12	119.1 (2)	C4—C5—O13	119.8 (2)
O11—C1—O12	127.5 (3)	C4—C5—O14	113.1 (2)
C1—C2—C3	114.7 (2)	O13—C5—O14	127.1 (3)
C1—C2—H7	108.98	O15—C6—O16	127.4 (3)
C3—C2—H7	110.16	C1—O11—H21	110.75
C1—C2—H8	104.24	C5—O14—H20	106.25
C3—C2—H8	110.07	C6—O16—Mg19	121.5 (2)
H7—C2—H8	108.42	C6—O16—Mg19^i^	135.7 (2)
C2—C3—C4	109.49 (13)	Mg19—O16—Mg19^i^	102.71 (11)
C2—C3—O17	110.0 (2)	C3—O17—H18	115.99
C4—C3—O17	112.0 (2)	O16—Mg19—O16^ii^	173.29 (19)
C3—C4—C5	118.9 (2)	O16—Mg19—O16^i^	77.29 (11)
C3—C4—H9	109.19	O16^ii^—Mg19—O16^i^	107.49 (12)
C5—C4—H9	106.41	O16—Mg19—O16^iii^	107.49 (12)
C3—C4—H10	109.34	O16^ii^—Mg19—O16^iii^	77.29 (11)
C5—C4—H10	106.05	O16^i^—Mg19—O16^iii^	92.92 (17)

Symmetry codes: (i) −*x*, −*y*+2, −*z*; (ii) −*x*, *y*, −*z*+1/2; (iii) *x*, −*y*+2, *z*+3/2.

### (II_DFT) Crystal data

**Table:**

C_12_H_12_MgO_14_	*c* = 5.924466 Å
*M**_r_* = 380.13	β = 82.5510°
Monoclinic, *C*2/*c*	*V* = 1500.25 Å^3^
*a* = 23.263806 Å	*Z* = 4
*b* = 10.977897 Å	

### (II_DFT) Data collection

**Table:**

*h* = →	*l* = →
*k* = →	

### (II_DFT) Fractional atomic coordinates and isotropic or equivalent isotropic displacement parameters (Å^2^)

**Table:**

	*x*	*y*	*z*	*U*_iso_*/*U*_eq_	
C1	0.066697	0.592918	0.019370	0.02800*	
C2	0.120835	0.663366	0.040473	0.01350*	
C3	0.111356	0.800364	0.083233	0.01350*	
C4	0.169510	0.865519	0.103042	0.01350*	
C5	0.206049	0.815740	0.276452	0.02800*	
C6	0.086358	0.858791	−0.123082	0.02800*	
H7	0.141872	0.622809	0.175820	0.017600*	
H8	0.149980	0.650794	−0.118440	0.017600*	
H9	0.196771	0.859661	−0.061861	0.017600*	
H10	0.161628	0.962195	0.135988	0.017600*	
O11	0.073635	0.474712	0.041921	0.02800*	
O12	0.020939	0.641776	−0.015759	0.02800*	
O13	0.210674	0.706317	0.318331	0.02800*	
O14	0.235389	0.900676	0.369735	0.02800*	
O15	0.112196	0.838454	−0.316242	0.02800*	
O16	0.041922	0.928258	−0.075928	0.02800*	
O17	0.069907	0.823253	0.279849	0.02800*	
H18	0.086850	0.823247	0.424908	0.036400*	
Mg19	0.00000	0.939597	0.25000	0.020200*	
H20	0.257581	0.861395	0.484930	0.036400*	
H21	0.036202	0.428868	0.030415	0.036400*	

### (II_DFT) Hydrogen-bond geometry (Å, º)

**Table:**

*D*—H···*A*	*D*—H	H···*A*	*D*···*A*	*D*—H···*A*
O11—H21···O12^i^	1.02	1.55	2.567	179
O14—H20···O13^ii^	1.01	1.64	2.640	176
O17—H18···O15^iii^	0.99	1.72	2.708	174
C4—H9···O13^iv^	1.10	2.57	3.580	152
C4—H10···O15^v^	1.09	2.47	3.522	161

Symmetry codes: (i) −*x*, −*y*+1, −*z*; (ii) −*x*+1/2, −*y*+3/2, −*z*+1; (iii) *x*, *y*, *z*+1; (iv) −*x*+1/2, −*y*+3/2, −*z*; (v) *x*, −*y*+2, *z*+1/2.

### (MGCITD_DFT) Crystal data

**Table:**

C_12_H_30_Mg_3_O_24_	*c* = 9.1350 Å
*M**_r_* = 631.05	β = 96.8600°
Monoclinic, *P*2_1_/*n*	*V* = 1226.25 Å^3^
*a* = 20.2220 Å	*Z* = 2
*b* = 6.6860 Å	

### (MGCITD_DFT) Data collection

**Table:**

*h* = →	*l* = →
*k* = →	

### (MGCITD_DFT) Fractional atomic coordinates and isotropic or equivalent isotropic displacement parameters (Å^2^)

**Table:**

	*x*	*y*	*z*	*U*_iso_*/*U*_eq_	
C1	0.32527	0.37608	−0.21401	0.00000*	
C2	0.38451	0.45577	−0.11329	0.00000*	
C3	0.36751	−0.34515	−0.03946	0.00000*	
C4	0.43213	−0.25338	0.03746	0.00000*	
C5	0.42398	−0.06235	0.12413	0.00000*	
C6	0.31593	−0.38215	0.07191	0.00000*	
H7	0.42506	0.48305	−0.17971	0.00000*	
H8	0.12316	0.09204	−0.06504	0.00000*	
H9	0.10594	0.28446	0.02046	0.00000*	
H10	−0.07582	0.31823	0.03007	0.00000*	
H11	−0.02225	0.33290	0.17064	0.00000*	
H12	0.13541	−0.44107	0.18391	0.00000*	
H13	0.18161	−0.39965	0.06432	0.00000*	
H14	0.40011	0.34753	−0.02685	0.00000*	
H15	0.46361	−0.21620	−0.04831	0.00000*	
H16	0.45875	−0.36309	0.11100	0.00000*	
H17	0.31484	−0.27005	−0.23049	0.00000*	
H18	0.21544	0.09673	0.18783	0.00000*	
H19	0.26158	0.27605	0.15127	0.00000*	
H20	0.03218	−0.25322	0.22859	0.00000*	
H21	0.08743	−0.08738	0.24278	0.00000*	
Mg22	0.00000	0.00000	0.00000	0.00000*	
Mg23	0.28120	0.00002	−0.03838	0.00000*	
O24	0.30983	0.47427	−0.33321	0.00000*	
O25	0.08758	0.15699	−0.01882	0.00000*	
O26	−0.04215	0.24084	0.09404	0.00000*	
O27	0.13785	−0.45485	0.07789	0.00000*	
O28	0.29211	0.23046	−0.17595	0.00000*	
O29	0.47379	−0.00062	0.20839	0.00000*	
O30	0.36791	0.02758	0.10966	0.00000*	
O31	0.26375	−0.27533	0.05738	0.00000*	
O32	0.32823	0.48746	0.17156	0.00000*	
O33	0.33975	−0.20083	−0.14661	0.00000*	
O34	0.23596	0.16252	0.10819	0.00000*	
O35	0.04053	−0.11092	0.20610	0.00000*	

### (MGCITD_DFT) Bond lengths (Å)

**Table:**

C1—C2	1.517	H6—O4	0.980
C1—O1	1.278	H7—O4	0.980
C1—O5	1.255	H11—O10	0.981
C2—C3^i^	1.549	H12—O11	0.983
C2—H1	1.092	H13—O11	0.975
C2—H8	1.090	H14—O12	0.992
C3—C2^ii^	1.549	H15—O12	0.980
C3—C4	1.536	Mg2—O1^iii^	2.069
C3—C6	1.561	Mg2—O5	2.017
C3—O10	1.440	Mg2—O7	2.091
C4—C5	1.522	Mg2—O8	2.086
C4—H9	1.096	Mg2—O10	2.112
C4—H10	1.092	Mg2—O11	2.026
C5—O6	1.262	O1—Mg2^iv^	2.069
C5—O7	1.276	O2—Mg1	2.083
C6—O8	1.268	O3—Mg1	2.057
C6—O9^ii^	1.264	O9—C6^i^	1.264
H2—O2	0.978	O12—Mg1	2.097
H3—O2	0.980	Mg1—O2^v^	2.083
H4—O3	0.989	Mg1—O3^v^	2.057
H5—O3	0.981	Mg1—O12^v^	2.097

Symmetry codes: (i) *x*, *y*+1, *z*; (ii) *x*, *y*−1, *z*; (iii) −*x*+1/2, *y*−1/2, −*z*−1/2; (iv) −*x*+1/2, *y*+1/2, −*z*−1/2; (v) −*x*, −*y*, −*z*.

### (MGCITD_DFT) Hydrogen-bond geometry (Å, º)

**Table:**

*D*—H···*A*	*D*—H	H···*A*	*D*···*A*	*D*—H···*A*
O35—H21···O32	0.980	1.868	2.831	171.6
O35—H20···O29	0.992	1.760	2.745	171.5
O34—H19···O32	0.975	1.947	2.877	158.8
O34—H18···O32	0.983	1.798	2.780	175.3
O33—H17···O24	0.981	1.947	2.783	141.5
O33—H17···O28	0.981	1.997	2.986	133.1
O27—H13···O31	0.980	1.865	2.841	173.3
O27—H12···O30	0.980	1.906	2.873	168.3
O26—H11···O29	0.981	1.778	2.750	169.8
O26—H10···O27	0.989	1.756	2.745	177.6
O25—H9···O27	0.980	1.910	2.887	174.0
O25—H8···O24	0.978	1.903	2.880	176.2
