# Supplementary material for: Crystal structures of two magnesium citrates from powder diffraction data
Source: Acta Crystallogr E Crystallogr Commun. 2020 Sep 8;76(Pt 10):1611–6. doi: 10.1107/S2056989020011913 (PMC7534234; doi:10.1107/S2056989020011913)
Supplement: Supplementary file 2 [file e-76-01611-sup2.docx]

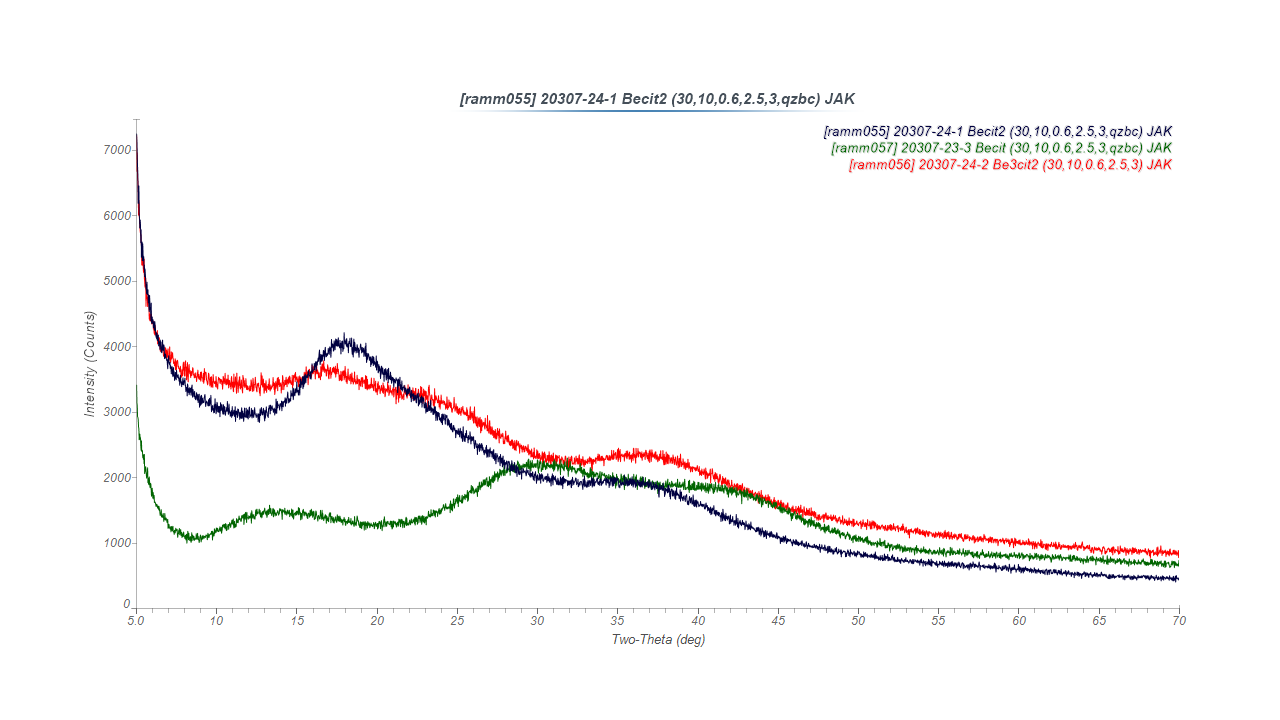


Figure S1: powder diffraction patterns of Be(H_2_C_6_H_5_O_7_)_2_ (black), Be(HC_6_H_5_O_7_) (green) and Be_3_(C_6_H_5_O_7_)_2_ (red), measured using Cu Kα radiation. The mass recoveries were 84.4, 98.0, and 110.0% respectively, so the compounds probably exhibit different states of hydration.
